# Surveillance of the Incidence of Non-Communicable Diseases (NCDs) with Sparse Resources: A Simulation Study Using Data from a National Diabetes Registry, Denmark, 1995–2004

**DOI:** 10.1371/journal.pone.0152046

**Published:** 2016-03-29

**Authors:** Ralph Brinks, Annika Hoyer, Sandra Landwehr

**Affiliations:** 1 German Diabetes Center, Institute for Biometry and Epidemiology, Duesseldorf, Germany; 2 University Hospital Duesseldorf, Hiller Research Unit for Rheumatology, Duesseldorf, Germany; 3 University Hospital Duesseldorf, Department for Statistics in Medicine, Duesseldorf, Germany; Public Health Agency of Canada, CANADA

## Abstract

We propose two new methods to estimate secular trends in the incidence of a chronic disease from a series of prevalence studies and mortality data. One method is a direct inversion formula, the second method is a least squares estimation. Both methods are validated in a simulation study based on data from a diabetes register. The results of the validation show that the proposed methods may be useful in epidemiological settings with sparse resources, where running a register or a series of follow-up studies is difficult or impossible.

## Introduction

In 2010, more than 65% of all global deaths were caused by non-communicable diseases (NCDs) such as cancer, cardiovascular and chronic respiratory diseases, diabetes and neurological disorders [1]. Compared to 1990, not only did the absolute number of NCD-attributable deaths rise from 27 to 35 million, but the proportion of deaths attributable to NCDs also rose (1990: 57%).

The enormous and increasing burden of NCDs have attracted the highest political councils, which, for instance, led to a resolution of the United Nations’ General Assembly [2]. The resolution demands strengthening of country-level surveillance systems for risk factors, determinants of health and health outcomes. This includes national surveillance of the prevalence and incidence of NCDs. Surveillance of prevalence alone is not always reasonable. The prevalence of a chronic disease may even increase though the incidence remains unchanged. The rise in prevalence may be due to improved survival of prevalent cases, which in turn may be due to improved disease treatment. Thus, surveillance of the *incidence* of NCDs is necessary as well. Secular trends of NCD incidences are especially important because they indicate changes in the risk profile of the population under consideration. Moreover, surveillance of temporal changes of the incidence may be helpful to judge effectiveness of population-based prevention programs, e.g., tobacco control programs in the US [3].

Common ways to detect secular trends in the incidence are to either performing a series of follow-up studies or to run a register. Both approaches may be expensive and time consuming. Furthermore, they both may lead to a variety of practical problems. As an example, consider a follow-up study where the people contracting the outcome, i.e., the cases, may feel stigmatized. This is likely to lead to a loss of follow-up among the the group of cases, possibly at a higher percentage than in the non-cases. In contrast, a series of cross-sectional studies can sometimes be easier to accomplish—and may lead to more valid estimates. An example of a periodically published summary of cross-sectional studies is the Diabetes Atlas of the International Diabetes Federation [4].

In [5] we described a method in which two cross-sectional studies combined with mortality information can be used to estimate the incidence of a chronic disease. Here we take this approach further and describe how a series of cross-sections can examine possible trends in the incidence of NCDs without the need to run a disease register and without a series of follow-up studies.

The next section introduces the necessary theoretical background for the methods. In the third section the theory is applied to data from a diabetes register in Denmark. The register observed an increasing incidence of diabetes between 1995 and 2004. We show that the trend is detectable using a sequence of prevalence and mortality data. The prevalence data can be obtained by cross-sectional studies and the mortality data may originate from official vital statistics and, for example, from case-control studies. The use of costly and lengthy follow-up studies is not always necessary.

## Illness-Death Model

**Fig 1:**
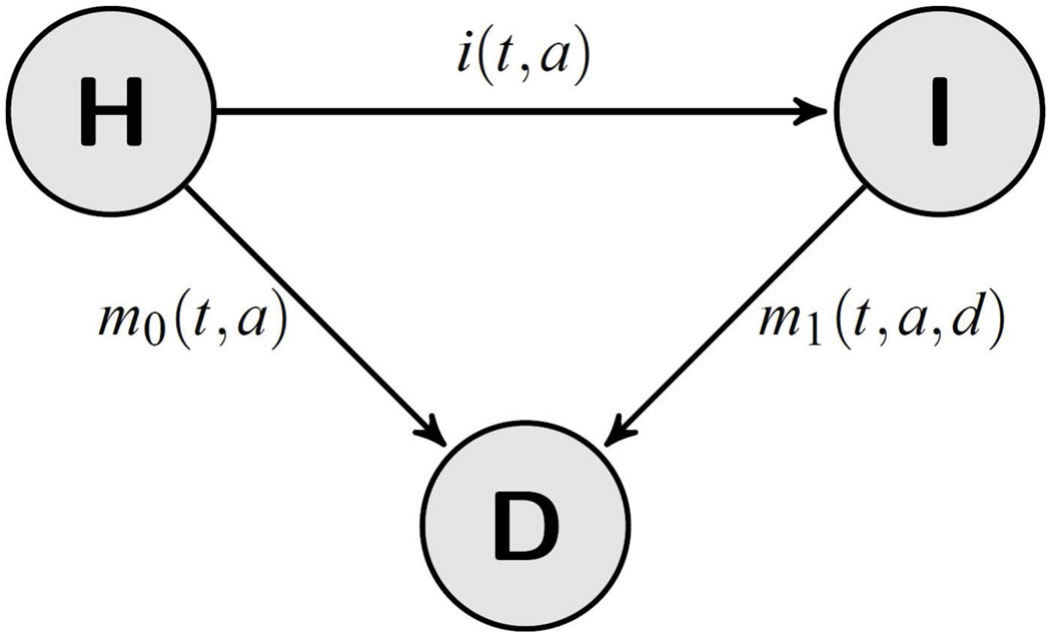
Chronic disease model with three states and the corresponding transition rates. People in the state *Healthy (H)* are healthy with respect to the disease under consideration. After onset of the disease, they transition to the state *Ill (I)*.

In modeling chronic (irreversible) diseases, the three-state model (compartment model) in Fig 1 is often used. This model is often called the *illness-death model* (IDM) [6] and dates back to at least 1951 [7]. The role of the IDM in biostatistics can hardly be valued enough. For example, it contributed to the development of the famous Kaplan-Meier estimator in survival and failure time analysis [8].

Let the number of persons in the states *Healthy (H)* and *Ill (I)* of the IDM be denoted by *S* and *C*, respectively. There are historical reasons for using the terminologies *S* (susceptibles) and *C* (cases). The transition intensities (synonym: rates) between the states are: the incidence rate *i* and the mortality rates *m*_0_ and *m*_1_ of healthy and diseased persons, respectively. These rates and the numbers *S* and *C* generally depend on the calendar time *t*, the age *a* and in the case of the mortality *m*_1_ also on the duration *d* of the disease. Although the following derivations hold true if *m*_1_ depends on *d* [5], for ease of notation we assume hereinafter that *m*_1_ is independent from *d*.

In [9] we showed how the rates *i*, *m*_0_, and *m*_1_, are related to the age-specific prevalence $p\left(t,a\right)=\frac{C(t,a)}{S(t,a)+C(t,a)}.$ If we consider only chronic diseases acquired after birth, i.e., *p*(*t*, 0) = 0 for all times *t*, then, for differentiable rates *i*, *m*_0_, and *m*_1_, the age-specific prevalence *p*(*t*, *a*) is the unique solution of a partial differential equation (PDE) and the associated initial condition:

$$\begin{array}{c} (\partial_{t}+\partial_{a})\;p=\left(1-p\right)\;\left(i-p\;\left(m_{1}-m_{0}\right)\right) \end{array}$$ (1)

$$\begin{array}{c} p(t,0)=0. \end{array}$$ (2)

Here, the notation $\partial_{x}=\frac{\partial }{\partial x},\;x\in \left\{t,a\right\},$ has been used. The partial differential Eqs (1) and (3) use the assumption that the persons who immigrate into or emigrate from the considered population have the same disease prevalence as the resident population. If this assumption does not hold true, the partial differential equation becomes slightly more complicated [9].

Typically, the mortality rate *m*_0_ for the healthy persons is not known, but the general mortality *m* = *pm*_1_ + (1 − *p*)*m*_0_, and the relative mortality $R=\frac{m_{1}}{m_{0}}$ are known. Therefore, Eq (1) becomes

$$\begin{array}{c} \left(\partial_{t}+\partial_{a}\right)\;p=\left(1-p\right)\;\left(i-m\;\frac{p\left(R-1\right)}{p\left(R-1\right)+1}\right). \end{array}$$ (3)

Note that the fraction on the right-hand side of the PDE Eq (3) is the *population attributable risk (PAR)*, which describes the proportion of deaths attributable to the chronic disease [10].

### Direct estimation of the age-specific incidence

Eqs (1) and (3) can be solved for the incidence rate. For *p*(*t*, *a*) ≠ 1 it holds

$$\begin{array}{cc} i(t,a) & =\frac{\left(\partial_{t}+\partial_{a}\right)\;p\left(t,a\right)}{1-p(t,a)}+p\left(t,a\right)\;\left(m_{1}\left(t,a\right)-m_{0}\left(t,a\right)\right)\\ & =\frac{\left(\partial_{t}+\partial_{a}\right)\;p\left(t,a\right)}{1-p(t,a)}+m\left(t,a\right)\;\frac{p\left(t,a\right)\left(R(t,a)-1\right)}{p\left(t,a\right)\left(R(t,a)-1\right)+1}. \end{array}$$ (4)

**Fig 2:**
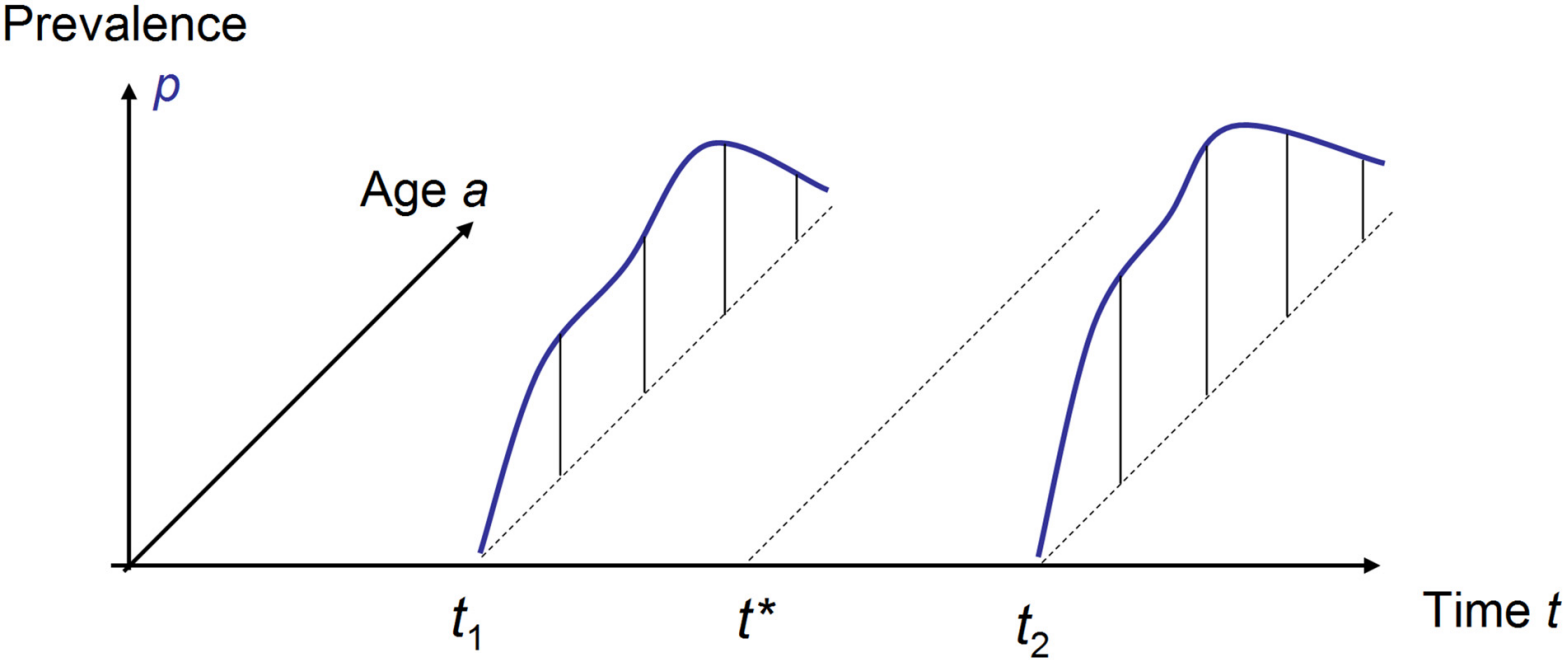
Two age courses of the prevalence surveyed at *t*_1_ and *t*_2_ are necessary to estimate the age-specific incidence at some point in time *t*^⋆^.

These equations have an important consequence: they make it possible to estimate the age-specific incidence rate from two prevalence studies. Consider the situation where the age-specific prevalence *p*(*t*, *a*) is given for two points in time *t* = *t*_1_ and *t* = *t*_2_, where *t*_1_ < *t*_2_. Assume further that the general mortality *m* and the relative mortality *R* are given at some time *t*^⋆^ where *t*_1_ < *t*^⋆^ < *t*_2_. This situation is depicted in Fig 2.

Then, Eq (4) allows estimating *i*(*t*^⋆^, *a*) [5]. Because Eq (4) follows directly from solving Eq (3) for *i*, we call this method the *direct estimation of the incidence*. In the next section we introduce a new alternative approach to estimate the incidence from two cross-sectional studies.

### Least squares estimation of the age-specific incidence

In epidemiological applications the *p*(*t*, *a*) are sampled at discrete ages *a*_*k*_, *k* = 1, …, *K*. Furthermore, we assume that we know the general mortality rate *m* and the relative mortality *R* at some time *t*^⋆^ where *t*_1_ < *t*^⋆^ < *t*_2_.

Typically, the *p*(*t*_*j*_, *a*_*k*_), *j* = 1, 2, *k* = 1, …, *K*, are subject to sampling uncertainty. Let *σ*_*jk*_ denote the standard error of *p*(*t*_*j*_, *a*_*k*_).

For a moment let us assume that we know *p* at *t*^⋆^, and that we have a “guess” of the age-specific incidence *i*^(*g*)^(*t*^⋆^, ⋅). Then, we can use the PDE Eq (3) to approximate *p* at *t*_2_ by

$$\begin{array}{c} p\left(t_{2},a|i^{\left(g\right)}\right)≐p\left(t^{⋆},a-h_{2}\right)+h_{2}\;\left(\partial_{t}+\partial_{a}\right)p\left(t^{⋆},a-h_{2}|i^{\left(g\right)}\right) \end{array}$$ (5)

where *h*_2_ = *t*_2_ − *t*^⋆^ and ≐ denotes the first order approximation. The values of the partial derivative (∂_*t*_ + ∂_*a*_)*p* are calculated by the right-hand side of the associated Eq (3).

Similarly, we can approximate *p* at *t*_1_:

$$\begin{array}{c} p\left(t_{1},a|i^{\left(g\right)}\right)≐p\left(t^{⋆},a-h_{1}\right)-h_{1}\;\left(\partial_{t}+\partial_{a}\right)p\left(t^{⋆},a-h_{1}|i^{\left(g\right)}\right) \end{array}$$ (6)

where *h*_1_ = *t*^⋆^ − *t*_1_.

As *i*^(*g*)^ was based on an initial guess, the calculated values for *p*(*t*_*j*_, *a*|*i*^(*g*)^), *j* = 1, 2, are likely to deviate from the measured values *p*(*t*_*j*_, *a*_*k*_).

Therefore, define the sum of the standardized squared error *X*^2^(*i*^(*g*)^) as

$$\begin{array}{c} \begin{array}{c} X^{2}\left(i^{\left(g\right)}\right)≔\sum_{j,k}\frac{{\left|p\left(t_{j},a_{k}\right)-p\left(t_{j},a_{k}|i^{\left(g\right)}\right)\right|}^{2}}{\sigma_{jk}^{2}}. \end{array} \end{array}$$ (7)

Then, estimation of the incidence *i* can be written in terms of a minimization problem:

$$\begin{array}{c} i=\arg\min_{i^{\left(g\right)}\geq 0}X^{2}\left(i^{\left(g\right)}\right). \end{array}$$ (8)

Thus, *i* is the weighted least squares solution, which minimizes the squared deviation between the estimated and measured *p* in *t*_1_ and *t*_2_. Underlying this minimization approach is the idea that the error *p*(*t*_*j*_, *a*_*k*_) − *p*(*t*_*j*_, *a*_*k*_|*i*^(*g*)^) is approximately normally distributed with a mean of zero and a standard deviation *σ*_*jk*_ [11].

So far, we have assumed that we know *p* at time *t*^⋆^, which is not the case if we have only data from two cross-sections at *t*_1_ and *t*_2_. In this case, we can approximate *p*(*t*^⋆^, *a*) using

$$p\left(t^{⋆},a\right)≐\frac{h_{2}}{h_{1}+h_{2}}p\left(t_{1},a-h_{1}\right)+\frac{h_{1}}{h_{1}+h_{2}}p_{k}\left(t_{2},a+h_{2}\right).$$

By applying the direct or the least squares estimators of the incidence consecutively for a series of cross-sections at *t*_1_ < *t*_2_ < ⋯ < *t*_*n*_, we are able to estimate trends in the incidence. This is shown in the next section.

## Validation of the Method: Diabetes in Denmark

Based on data from the Danish Diabetes Register, we first simulate a sequence of cross-sectional studies for the years 1995–2004. Next, we estimate the age-specific incidence rates by repetitively applying the methods described above to the simulated prevalence data. We assume that the general mortality in the population (*m*) and the relative mortality $R=\frac{m_{1}}{m_{0}}$ are known. Finally, the incidence rate used as input for the simulation—we will call it the “true incidence”—and the estimated incidence are compared in terms of the absolute relative error.

### Simulation of a series of cross-sections

The data used for the simulation are based on a complete survey of physician diagnosed diabetes (type 1 and type 2 diabetes) in the Danish population (*n* > 5 million) [12]. For this validation task we confine ourselves to the male population aged ≥ 30. We do not use individual-level data, only (aggregated) rate and prevalence data as published in [12]. The starting point is the prevalence of diabetes on January 1, 1995. Due to the imposed age restriction, the initial condition Eq (2) changes to:

$$\begin{array}{c} p\left(1995,a\right)=p_{0}\left(a\right),\;a\geq 30, \end{array}$$ (9)

where *p*_0_ is the age-specific prevalence in 1995.

The incidence rate for 2004 is described in detail for all age groups in the article by Carstensen et al. [12]. For the years 1995–2003 the authors report a yearly increase of 5.3% per year (relative to the rate in 2004). This means that in 2003, the incidence rate was 95.0% (1/1.053) of the rate in 2004. The growth rate of 5.3% per year is an average across all age groups. Similarly, the mortality rates *m*_0_ and *m*_1_ of the non-diabetic and diabetic male population, respectively, are reported for 2004. The average reductions for *m*_0_ and *m*_1_ are 2.5% and 3.9% per year, respectively. In simulating the prevalence, we approximate the rates reported in 2004 and assume that the reported annual changes hold for *all* ages ≥ 30. This is necessary because the age-specific change rates are not reported. Further details are provided in S1 Text.

Having the initial condition and the rates *i*, *m*_0_ and *m*_1_ at hand, we solve the PDE Eq (1) with initial condition Eq (9) by numerical integration. This is performed by a Runge-Kutta method (routine rk4 in the deSolve package for the statistical software R). As result we obtain the age-specific prevalences *p*(1996, *a*), *p*(1997, *a*), …, *p*(2004, *a*).

**Fig 3:**
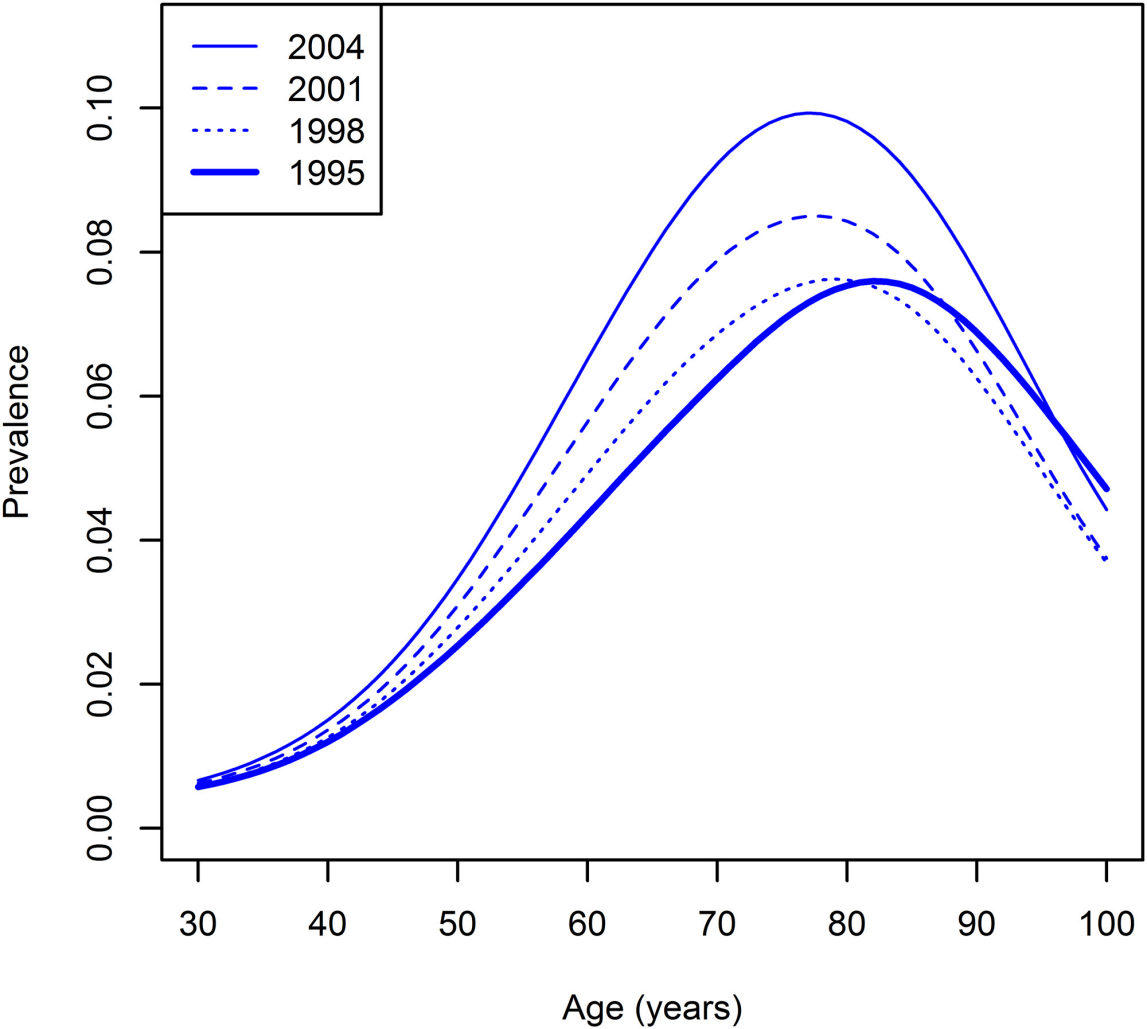
Age-specific prevalences for the years 1995, 1998, 2001, and 2004.

Fig 3 shows the age-specific prevalences for the years 1995, 1998, 2001, and 2004. Because the data for 1995 were used as the initial condition it is shown as solid line.

### Estimating the incidence from the cross-sections

After calculating the age course of the prevalences in the years 1996–2004, we estimate the mid-year age-specific incidence (denoted *t* = 1995.5, 1996.5, …, 2003.5) by successively applying Eqs (4) and (8) to pairs of consecutive years. For example, the incidence for *t* = 2003.5 is estimated by the age-specific prevalences in 2003 and 2004.

The mortality data (*m*, *R*) necessary for the application of Eqs (4) and (8) are assumed to be known for the validation study. We can calculate the relative mortality *R* by $R=\frac{m_{1}}{m_{0}}$ and the mortality *m* of the general population by *m* = *pm*_1_ + (1 − *p*)*m*_0_, and use these as the input for the algorithms.

**Fig 4:**
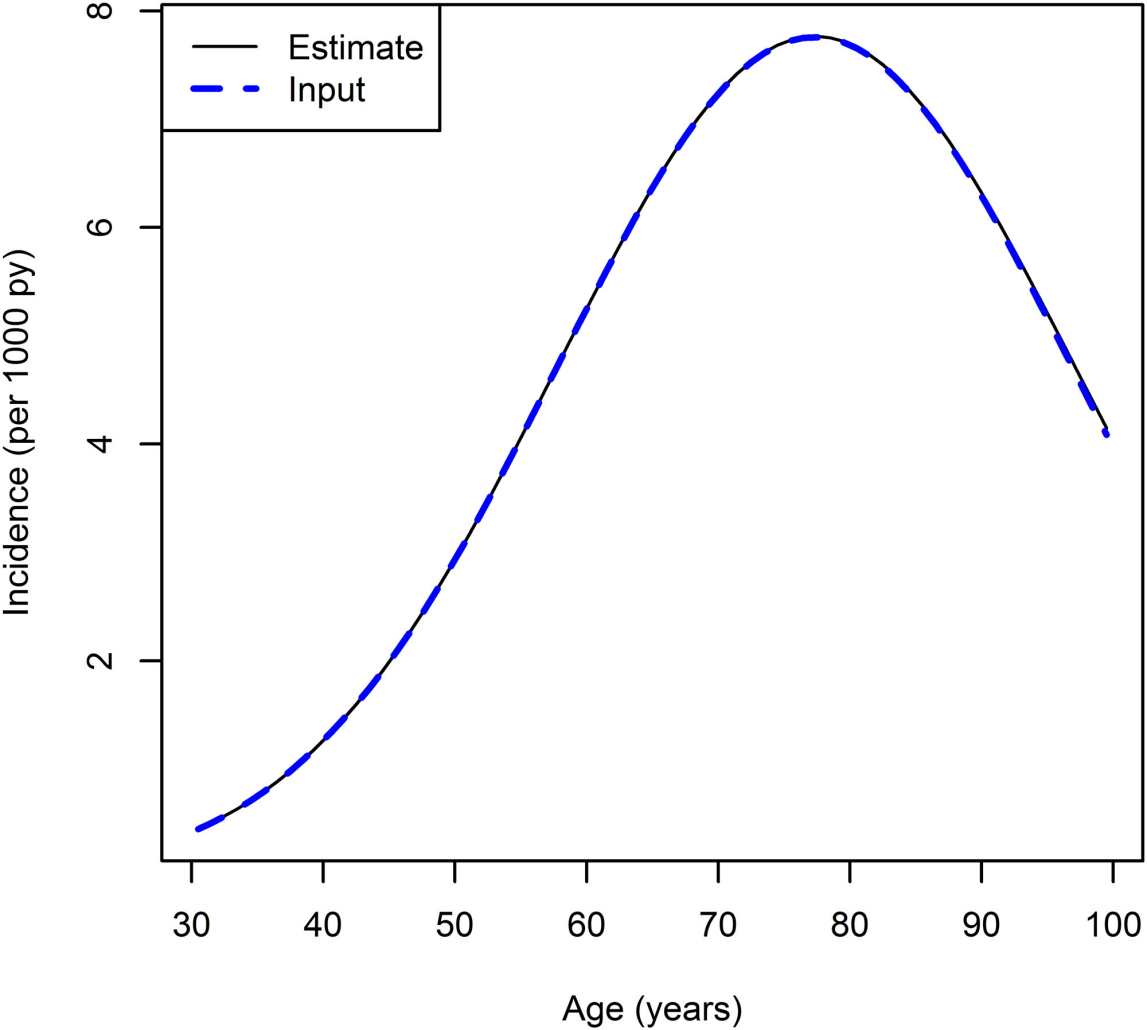
Age-specific incidence rate for *t* = 1995.5: true (dashed line) and estimated incidence (solid).

Fig 4 shows the estimated age-specific incidence for *t* = 1995.5 compared to the “true incidence”, which was used as input for the simulation. Both methods, direct estimation and least squares estimation (with *σ*_*jk*_ = 1), yield the same results. Visually, the curves are in nearly perfect agreement.

To quantify the deviation between the “true” and the estimated incidence, we calculate the absolute relative error. For each of the yearly estimates from 1995.5 to 2003.5, the median and maximum relative error over all ages *a* = 30.5, 31.5, …, 99.5 are calculated. The results are shown in Table 1. Note that the units in Table 1 are per 100,000, which means that the overall maximum of the absolute relative error is below 0.9 percent.

**Table 1:** Absolute relative errors of the incidence estimates (per 10^5^). Median and maximum refer to all age-specific estimates for the corresponding point in time (*t*^⋆^).

	Direct method		Least squares method (*σ*_*jk*_ = 1)	
Time *t*^⋆^	Median	Maximum	Median	Maximum
1995.5	97	819	97	819
1996.5	94	658	94	658
1997.5	91	550	91	550
1998.5	88	478	88	478
1999.5	85	428	85	428
2000.5	83	394	83	394
2001.5	81	371	81	371
2002.5	79	354	79	354
2003.5	77	343	77	343

If we compare the results of the direct and the least squares method in Table 1, we see that both methods lead to the same relative errors. Indeed, both methods yield very similar (but not equal) estimates for the age-specific incidence. There are two reasons for the similarity. First, there is no sampling uncertainty. As the main difference between the methods is the possible weighting of the prevalences with different sampling uncertainties (measured by *σ*^2^ in Eq (7)), both methods should yield similar results. Second, the prevalence is sampled in steps of one year in the age- and time-dimension. Hence, the impact of the discretization errors of the partial derivative (∂_*t*_ + ∂_*a*_)*p* in Eqs (4), (5) and (6) is small.

**Fig 5:**
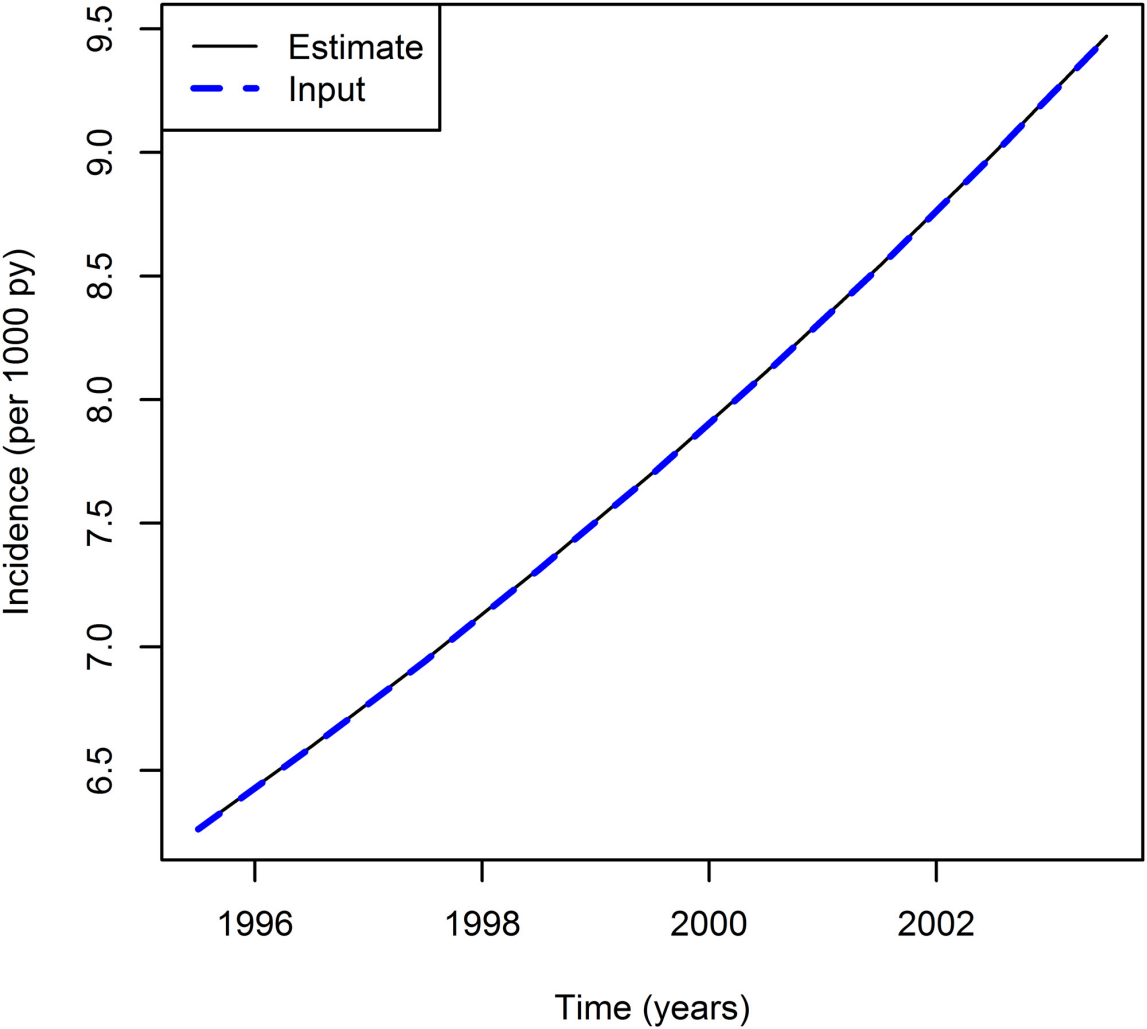
Incidence rate for the age group *a* = 65.5 over calendar time *t*: true (dashed line) and estimated incidence (solid line).

The incidence chosen as input data increases by 5.3% per year. Now, we examine whether this trend can be detected by the estimation methods. For illustration, we compare the data for age *a* = 65.5. Fig 5 shows the estimated incidence and the true incidence for *t* = 1995.5, 1996.5, …, 2003.5. Again, both methods direct and least squares estimates yield the same results—visually there is no difference between the two curves.

Hence, we may conclude that the estimation method is capable of accurately detecting the trend in the incidence—just by using the data from the prevalence studies and the mortality.

### Estimating the incidence in the presence of sampling error

So far, the sampling errors *σ*_*jk*_ were assumed to be equal. This means that each measurement *p*(*t*_*j*_, *a*_*k*_) gets the same weight when minimizing *X*^2^ in Eq (7). However, in real surveys the sampling of the age groups may be different. In a representative sample of a population, for instance, the sampling scheme is chosen with respect to the age distribution of the underlying population. This may lead to *σ*_*jk*_ being different from each other. An example is given as part from S1 File (TestCase1_A). In this test case we assume age groups of length five years with a decreasing sample size in the higher age groups. The age-specific prevalence is calculated as in the previous section. Then, additive binomial noise is superimposed on the calculated prevalence to mimic sampling uncertainty. The additive binomial noise for year *j* and age group *k* is assumed to have a mean of zero and the variance given by:

$$\begin{array}{c} \sigma_{jk}^{2}=\frac{\left[1-p\left(t_{j},a_{k}\right)\right]\;p\left(t_{j},a_{k}\right)}{n_{jk}}. \end{array}$$ (10)

**Fig 6:**
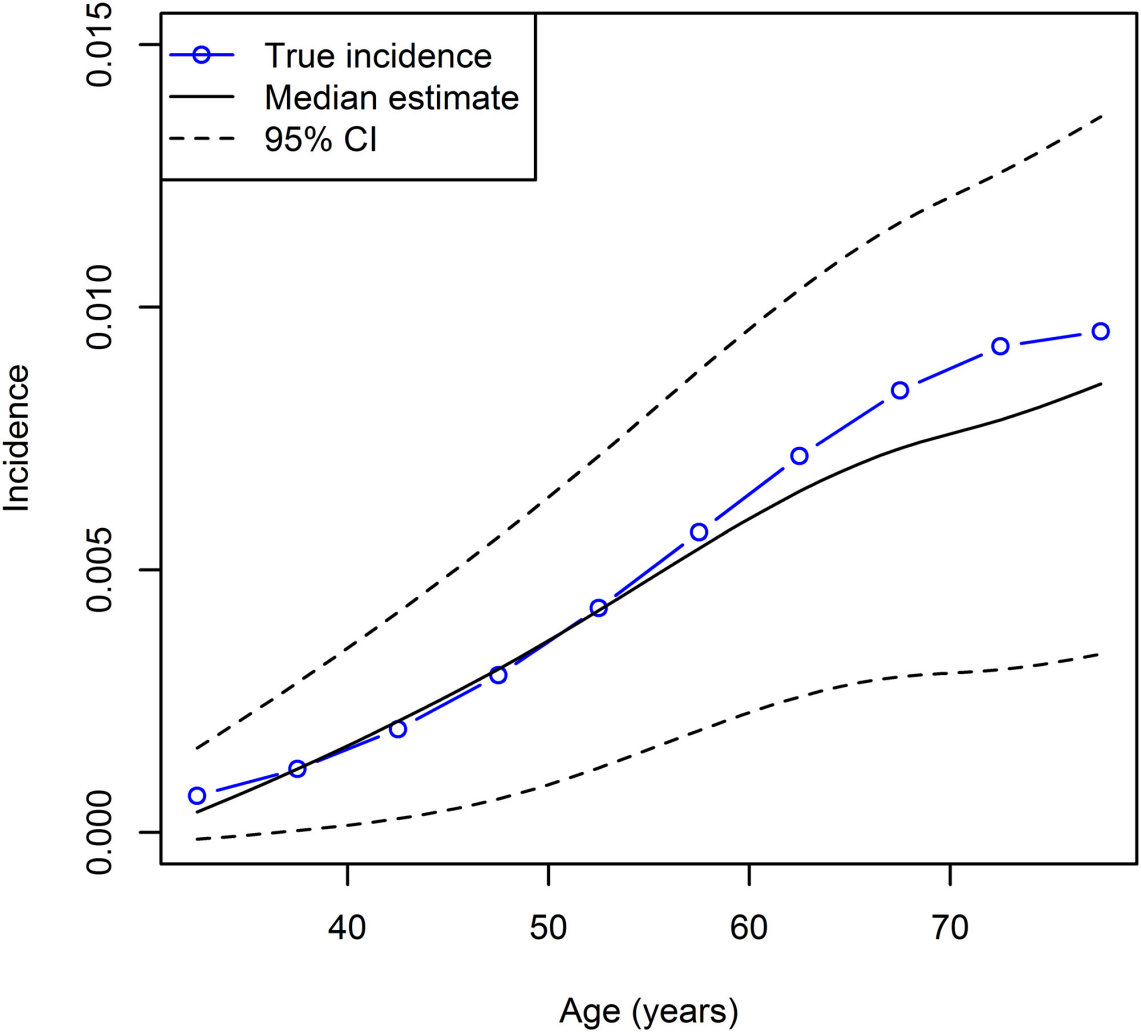
Age-specific incidence rates for *t* = 1996.5 based on the direct estimation and *B* = 2000 bootstraps. True (blue line) and estimated incidence (black lines): median (solid line) and 95% confidence bounds (dashed).

Then, the prevalence distorted by the additive noise serves as an input for the estimation method. To cope with the sampling uncertainty, a bootstrap technique is applied [13]. For each of *B* = 2000 bootstraps with binomial noise, we apply the direct incidence estimation. If we then plot the estimated incidences, we obtain the result shown in Fig 6. The true incidence (blue line) is compared to the median of the 2000 bootstrap estimates (solid black line) and the 95% confidence bounds (dashed black line).

For more details and further test cases the reader may refer to S2 Text and S1 File.

## Discussion and Conclusions

This work presents two novel methods for deriving trends in incidence from a sequence of prevalence studies are presented. The first method, called *direct estimation* is based on repetitively applying a previously published algorithm [5]. The second method is based on a least squares approach and has not been published previously. Both methods lead to the same results when perfect measurements (i.e., those without sampling uncertainties) are assumed. However, real surveys typically face sampling uncertainties. These uncertainties may be included into the least squares estimation, giving more weight to those measurements that have a higher accuracy. Alternatively, direct estimation can address statistical uncertainties by using resampling techniques as described in the previous paragraph.

Both methods, the direct and the least squares estimation, are based on a differential equation, which has shown superiority over other approaches for estimating the incidence from prevalence data [14]. The differential equation used in this work does not depend on assumptions about the transition rates. Hence, the estimation methods may be called *non-parametric*. The only (implicit) assumption is that the people who immigrate into or emigrate from the considered population have the same prevalence as the resident population. In situations where this assumption is violated, the differential equation must take additional terms for migration into account [9].

A simulation study based on the data from the Danish National Diabetes Register shows that both methods provide very accurate estimates of the age-specific incidence rates for a sequence of cross-sectional studies. This makes it possible to derive secular trends in the incidence. Incidence trends are especially important because they can indicate substantial changes of the risk profile in the population under consideration. With a view to the tremendous effort required to collect incidence data, this method may offer an alternative to the costly follow-up studies that would otherwise be required.

Apart from the sequence of prevalence studies, the new method requires age- and time-specific mortality data. First, we need the overall mortality for the population. Second, we need the relative mortality. While the overall mortality is often available from official vital statistics or death registries, the relative mortality, i.e. the ratio of the mortality rates of healthy and diseased persons, requires evidence from epidemiological studies. For example, case-control studies are capable of estimating the relative mortality [15]. Similarly, nested case-control studies can be used for this purpose [16]. Case-control studies provide an effective opportunity to estimate the relative mortality, because they can be conducted with relatively low effort compared to other study types [10]. Although the partial differential equation the estimation methods are based upon can be formulated in terms of the excess mortality (*m*_1_ − *m*_0_, see Eq (1)), the equivalent differential Eq (3) in terms of the mortality rate ratio (*m*_1_/*m*_0_) and the overall mortality (*m*) has practical advantages: Rate ratios are more likely to be generalizable from one population to another than rate differences [17]. Furthermore, estimates of the overall mortality in a population, i.e., the general mortality, are more easily obtainable than disease-specific death rates (such as *m*_1_). Note that all mortality rates in this work do not take the cause of death into account. The rates refer to all-cause mortality in a specific subset of the population. For instance, *m*_0_ is the overall mortality rate in the non-diabetic population.

Several extensions and modifications of the methods are possible. Changes in the age sampling can easily be made to the provided R scripts in S1 File. There are no restrictions on high or low age groups. Differences in covariates such as sex or education may be treated by stratification. The inclusion of covariates directly into the differential equations is subject to further research and is beyond the scope of this article.

Although the validation study uses data about diabetes, the methods are applicable to any disease where the underlying illness-death model in Fig 1 is able to capture important aspects of the disease. These comprise all diseases with incomplete cures, i.e., an elevated mortality once having contracted the disease. Examples are cardiovascular disease, cancer and rheumatic diseases. The model may be useful even in infectious diseases with incomplete cures, such as HIV.

A strength of the method proposed in this manuscript is that it does not require individual data, for example, the time of onset of a chronic disease. If these additional data are available, e.g., from electronic health records, other methods are preferable as summarized in the review of Keiding [18].

Although the treatment and impact of uncertainty in the prevalence data is discussed extensively in this article, a limitation may be seen in the fact that we have not considered the effect of uncertainty or errors in the mortality data. Studying the impact of errors in the mortality data and their interplay with errors in the prevalence data on the incidence estimates is subject to future work.

A typical application of the method is the conversion of a sequence of cross-sectional surveys that aimed to examine the age-specific prevalence of a chronic disease (or another irreversible health related state) into a sequence of incidence data. The method requires relatively low effort and therefore may be useful in settings with sparse resources.

## Supporting Information

S1 Text Detailed description of generating the input data for the validation. (DOC)

S2 Text Description of the source code. To allow independent validation of the results of this article and to advocate the reproducibility of algorithms and computational experiments, the scripts that produced the results of this article are available as supporting information. In addition, scripts for generating test data and making estimates from a sequence of prevalence studies are provided. All scripts can be used with the free statistical software R (The R Foundation for Statistical Computing).
(DOC)

S1 File Source code. The zip file contains the source files as described in S2 Text. After unzipping please refer to the readme.txt file.
(ZIP)
