# Supplementary material for: Surveillance of the Incidence of Non-Communicable Diseases (NCDs) with Sparse Resources: A Simulation Study Using Data from a National Diabetes Registry, Denmark, 1995–2004
Source: PLoS One. 2016 Mar 29;11(3):e0152046. doi: 10.1371/journal.pone.0152046 (PMC4811427; doi:10.1371/journal.pone.0152046)
Supplement: S1 Text — (DOC) [file pone.0152046.s001.doc]

**Detailed information of simulating a series of cross-sections for the validation study**

Supporting Information S1 to *Surveilance of the Incidence of Non-Communicable Diseases (NCDs) with Low Resources* by Ralph Brinks, Annika Hoyer, and Sandra Landwehr

To generate the prevalence data in the series of cross-sections, the partial differential equation (PDE) (1) with the initial condition (9) is numerically solved. The PDE requires three rates as input, the incidence rate *i* and the mortality rates *m*_0_ and *m*_1_. Those were chosen to be the approximated rates of the male persons in the Danish National Diabetes Register (Carstensen et al. 2008).

Incidence rate

From Figure 1 in Carstensen et al. 2008 we observe that the logarithm of the age-specific incidence rate of diabetes in the male population in 2004 is approximately parabolic for *a* ≥ 30 (blue curve). By visually inspecting the graph, we chose the logarithm of the incidence rate in 2004 to be the least-squares parabola defined by the points

[*a*, *log*(*i*(2004, *a*))] = {[30, *log*(0.0065], [40, *log*(0.00195)], [50, *log*(0.0045)], [60, *log*(0.0085)], [70, *log*(0.0112)], [80, *log*(0.0117)], [90, *log*(0.0095)], [100, *log*(0.0063)]}. The logarithm *log* is the natural logarithm to the basis *e* = 2.71828... We first fit the parabola and then take the anti-logarithm (*exp*) to get the incidence rate. Figure S1.1 shows the resulting age-specific incidence rate in 2004.

Figure S1.1 Age-specific incidence rate in the year 2004.

To account for the annual trend in the incidence (5.3% per year, Carstensen et al. 2008), we multiply by 1.053 for each year increase in calendar time *t*.

Mortality rates:

From Figure 4 in Carstensen et al. 2008 we observe that the logarithm of the age-specific mortality rate *m*_0_ in the non-diabetic male population in 2004 is approximately parabolic for *a* ≥ 40 (blue dotted curve). Similarly, the logarithm of the mortality of the Danish men with diabetes is linear for *a* ≥ 60 (blue solid curve). From visually inspecting these graphs, we fit straight lines and extrapolate the lines to ages below 40 and 60, respectively. The exact values that were used to fit the lines are given in Supporting Information S2. The resulting mortality rates are shown in Figure S1.2

Figure S2.2 Age-specific mortality rates in the year 2004.

The deviations from the straight lines in the younger age groups are negligible for our example, because in younger ages the mortality is relatively low and does not have a high impact on modelling the prevalence in the PDE (1). Also consider the general note below.

General note: we just use approximations to the rates reported in Carstensen et al. 2008. These rates do not claim to be the best possible approximations. They were just used to generate plausible and realistic prevalence data in the simulated series of the cross-sections. The prevalence data were then chosen as input for the estimation methods (direct and least squares). To judge about the quality of the estimated incidence rate, the estimated values were compared to the approximated incidence rate.
