# Supplementary material for: Surveillance of the Incidence of Non-Communicable Diseases (NCDs) with Sparse Resources: A Simulation Study Using Data from a National Diabetes Registry, Denmark, 1995–2004
Source: PLoS One. 2016 Mar 29;11(3):e0152046. doi: 10.1371/journal.pone.0152046 (PMC4811427; doi:10.1371/journal.pone.0152046)
Supplement: S2 Text — To allow independent validation of the results of this article and to advocate the reproducibility of algorithms and computational experiments, the scripts that produced the results of this article are available as supporting information. In addition, scripts for generating test data and making estimates from a sequence of prevalence studies are provided. All scripts can be used with the free statistical software R (The R Foundation for Statistical Computing). (DOC) [file pone.0152046.s002.doc]

**Detailed information about the use of estimation tool**

Supporting Information S2 to *Surveilance of the Incidence of Non-Communicable Diseases (NCDs) with Low Resources* by Ralph Brinks, Annika Hoyer, and Sandra Landwehr

**Format of the input data**


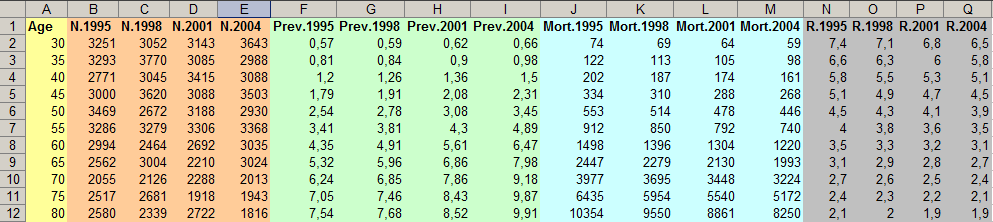


The estimation tool (run with the R-script "estimateIncidence.R") requires the input data to be arranged in five blocks. For illustration, in the figure above these blocks are colored in yellow, orange, green, blue and grey.

The first block (yellow) always contains one column (column A in the figure). This is the age to which the data of the corresponding row is referring to. Typically this is the middle of the associated age group (30, 35, ..., 80 in the figure). Problems may occur at the lower and upper end of the age sampling. For example, the highest age group in an epidemiological study may be something like 80+, which means '80 years of age or older', then the average age in this age class should be used.

The remaining blocks (orange, green, blue, grey) comprise as many columns as cross-sections are available. In the example (see figure), there are four cross-sections (1995, 1998, 2001 and 2004 as indicated in the captions in the first row). Note, that the years when the data of the cross-sections were surveyed are not read from the caption in the first row (these are just for explanation purposes), but from line 25 in "estimateIncidence.R" script

e.years <- c(1995, 1998, 2001, 2004)

This line is likely to be modified by the user when the estimation tool is run with own data.

The second block (orange) always contains the numbers of persons in the age groups whose mid points were given in column A. The third block (green) comprises the prevalence data (in percent) and the fourth block is the general mortality rate (per 100,000). The last block (grey) represents the relative mortality.

Note that the order of the columns within each of the second to fifth block needs to refer to the same cross-section. For instance, the second column of each of these blocks must refer to the second cross-section (1998 in the example).

**Sampling uncertainty**

To treat the statistical uncertainty in the cross-sectional data, a bootstrap resampling technique has been implemented. The numbers N of persons in the age strata (orange block, see above) are used to mimic binomial noise with mean zero and standard error SE = p (1 - p)/N. Based on 2000 bootstraps, it is investigated how this sampling uncertainty in the prevalence data propagates into the estimate of the incidence. The lines of code in the lower part of the script "estimateIncidence.R" are devoted to this. The number of bootstraps may be modified. The default value of 2000 bootstraps requires less than three seconds on an Intel i3, 3.3 GHz desktop personal computer with 8 GB RAM.

Trying different numbers N of persons in the age strata allows to make an estimate about the sample size or the width of the confidence bounds in the different age groups.

**Overview of the provided test cases**

Four test cases are provided with this work. The input data are provided in the files (TestDataX_Y.csv, X = 0,1 and Y = A, B). The value X = 0, 1 indicates if binomial noise has been added to the calculated prevalence (0 = no, 1 = yes).

Y refers to the age sampling. Age-sampling A has a regular sampling schema (age group midpoints every five years), while sampling B has age group midpoints of five and ten years difference.

The R-script "estimateIncidence.R" reads in one of the testdata-sets and provides the incidence estimate based on the prevalence and mortality data as described above.

All the testdata "TestDataX_Y.csv" have been generated from the R-script "generateTestData.R". This R-script may be used to generate own test data sets.
